# A new technique for bedside placement of enteral feeding tubes: a prospective cohort study

**DOI:** 10.1186/cc9407

**Published:** 2011-01-07

**Authors:** Günther Zick, Alexander Frerichs, Markus Ahrens, Bodo Schniewind, Gunnar Elke, Dirk Schädler, Inéz Frerichs, Markus Steinfath, Norbert Weiler

**Affiliations:** 1 Department of Anaesthesiology and Intensive Care Medicine, University Medical Centre Schleswig-Holstein, Campus Kiel, Arnold-Heller-Straße, 24105 Kiel, Germany; 2 Department of General Surgery and Thoracic Surgery, University Medical Centre Schleswig-Holstein, Campus Kiel, Arnold-Heller-Straße, 24105 Kiel, Germany

## Abstract

**Introduction:**

To accomplish early enteral feeding in the critically ill patient a new transnasal endoscopic approach to the placement of postpyloric feeding tubes by intensive care physicians was evaluated.

**Methods:**

This was a prospective cohort study in 27 critically ill patients subjected to transnasal endoscopy and intubation of the pylorus. Attending intensive care physicians were trained in the handling of the new endoscope for transnasal gastroenteroscopy for two days. A jejunal feeding tube was advanced via the instrument channel and the correct position assessed by contrast radiography. The primary outcome measure was successful postpyloric placement of the tube. Secondary outcome measures were time needed for the placement, complications such as bleeding and formation of loops, and the score of the placement difficulty graded from 1 (easy) to 4 (difficult). Data are given as mean values and standard deviation.

**Results:**

Out of 34 attempted jejunal tube placements, 28 tubes (82%) were placed correctly in the jejunum. The duration of the procedure was 28 ± 12 minutes. The difficulty of the tube placement was judged as follows: grade 1: 17 patients, grade 2: 8 patients, grade 3: 7 patients, grade 4: 2 patients. In three cases, the tube position was incorrect, and in another three cases, the procedure had to be aborted. In one patient bleeding occurred that required no further treatment.

**Conclusions:**

Fast and reliable transnasal insertion of postpyloric feeding tubes can be accomplished by trained intensive care physicians at the bedside using the presented procedure. This new technique may facilitate early initiation of enteral feeding in intensive care patients.

## Introduction

Feeding the critically ill patient should be preferentially accomplished via the enteral route [1,2]. A recent meta-analysis revealed that mortality and the incidence of pneumonia were significantly reduced in patients with enteral nutrition within 24 hours [3]. Parenteral nutrition may be associated with higher mortality [4].

Intolerance of gastric feeding and high gastric volumes are the main obstacles for enteral nutrition [5]. If intragastric feeding fails despite prokinetic therapy with erythromycin and metoclopramide it is recommended to place a feeding tube into the jejunum without delay. The advantages of postpyloric feeding are a lower incidence of regurgitation and microaspiration and improved tolerance of enteral nutrition [6-8].

Various methods of endoscopic placement of nasoenteral feeding tubes exist [9]. The standard bedside procedure requires transoral endoscopy. Another method introduces the tube through the instrument channel of the endoscope with subsequent transfer from the oral to the nasal cavity [10]. These procedures usually are performed by an experienced endoscopist. When the endoscopist is not available the recommended start of enteral nutrition within the first 24 hours may be delayed. Self-advancing tubes could be an alternative; however, the correct placement of these tubes may take a long time [11].

**Figure 1:**
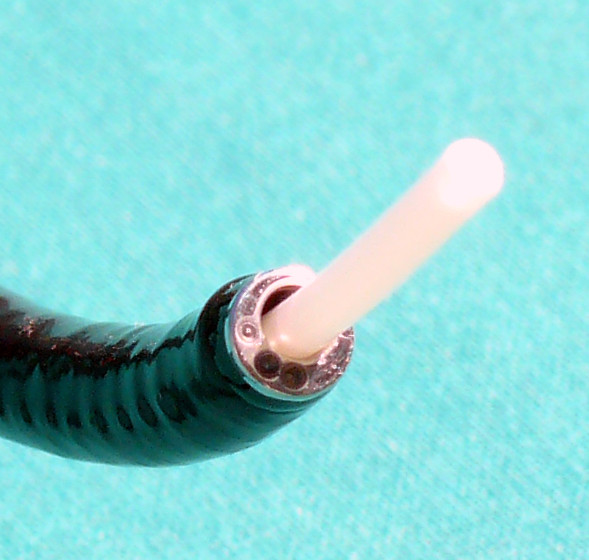
**Tip of endoscope, instrument channel and indwelling feeding tube**. Endoscope with an outer diameter of 6.0 mm and an instrument channel of 3.2 mm with the intestinal feeding tube exiting the instrument channel.

To solve these problems and to provide the intensive care unit (ICU) physician with an easy bedside method for rapid placement of feeding tubes, a new endoscope was developed. It can be introduced nasally and has an instrument channel large enough to accommodate the tube for enteral nutrition (Figure 1). The reduced diameter is associated with reduced optical quality and steering capabilities; however, this renders the handling of the new endoscope similar to a bronchoscope and is more familiar to an ICU physician.

The goal of this prospective cohort study was to evaluate whether ICU physicians were able to reliably insert a postpyloric feeding tube using this new endoscope at the bedside after a short training period.

## Materials and methods

The study was performed with approval of the ethics committee of the Christian Albrechts University Kiel in two surgical ICUs of the University Medical Center Schleswig-Holstein, Campus Kiel, Kiel, Germany. The need for informed consent was waived by the ethics committee.

An endoscope with an outer diameter of 6.0 mm, an instrument channel of 3.2 mm and a working length of 1,500 mm was used (FSB-18V, Pentax, Hamburg, Germany). A camera monitor system (AIDA DVD, Storz, Tuttlingen, Germany) was connected with an adapter (29020, Karl Storz, Tuttlingen, Germany). 8 Fr (2.7 mm) intestinal feeding tubes with a length of 4,000 mm were used in combination with 16 Fr gastric tubes of 1,000 mm (BCD 22 to 400 cm, Fresenius Kabi, Bad Homburg, Germany).

Patients with an indication for enteral nutrition therapy and high gastric volumes despite medication with metoclopramide and erythromycin were included in the study. Exclusion criteria were contraindications to enteral nutrition (for example, obstruction of the passage after trauma or surgery) or patients with a prior history of upper gastrointestinal bleeding.

A team consisting of an ICU physician and an endoscopist were trained by the manufacturer for two days. The tube placements were performed by the intensivist. The endoscopist supervised the first 10 placements.

**Figure 2:**
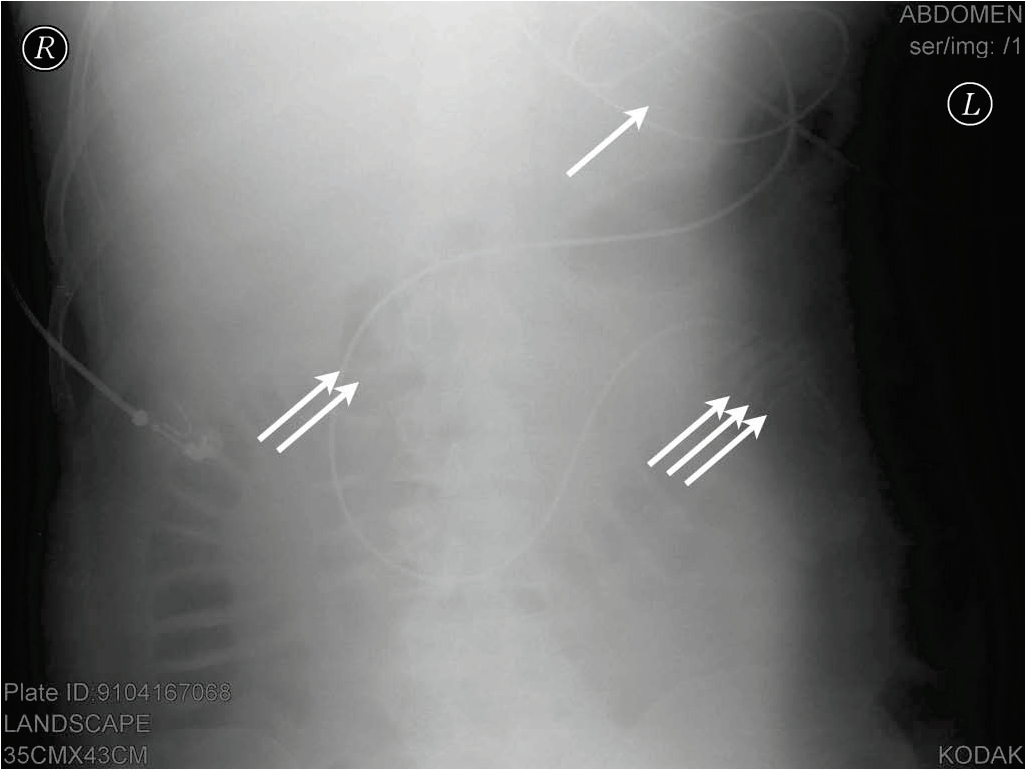
**Abdominal X-ray showing the position of the feeding tube**. Abdominal X-ray examination after the placement of the feeding tube in patient 8. Loop formation in the stomach (one arrow), the location of the tube in the duodenum (two arrows) and the contrast medium lining the jejunal wall (three arrows) are indicated.

All endoscopies were performed at the bedside. The patients were sedated, intubated and mechanically ventilated. The endoscope was inserted into the nose and continuously advanced through the oesophagus and stomach under visual control. Then the pylorus was intubated and the endoscope placed in the jejunum. The feeding tube was advanced via the instrument channel and its tip positioned in the jejunum. Afterwards, the endoscope was removed while the feeding tube was advanced through the instrument channel at the same rate. In order to relieve high gastric residual volumes a second tube was positioned in the stomach over the first one. After the procedure was completed, an X-ray examination with a contrast agent was performed to check the correct position (Figure 2).

Primary outcome was the successful jejunal placement of the tube. Secondary outcomes were time needed for the placement, complications like bleeding and formation of loops, and the assessment of the placement difficulty using a score (grade 1: easy to grade 4: difficult). Data are given as means and standard deviations.

## Results

From July 2008 to August 2009, 34 jejunal tube placements were performed with the described technique in 27 patients. Patients' characteristics are presented in Table 1.

**Table 1:** Patients' characteristics

Patients (no.)	27
Age (years)	66 ± 16
Gender (no.)	
Male	16
Female	11
SAPS II score^1^	44 ± 13
Diagnosis (no.)	
Abdominal/liver surgery	8
Trauma	5
Pancreatitis	4
Aortic disease/surgery	3
Cardiac surgery	2
Intracranial bleeding	2
Others	3

^1^SAPS: simplified acute physiology score. Data are shown as absolute numbers or mean values.

± standard deviation.

The placement procedure lasted 28 ± 12 minutes. The following difficulty scores were obtained: grade 1: 17 patients; grade 2: 8 patients; grade 3: 7 patients; grade 4: 2 patients. Repeated placement was performed in seven cases and resulted from tube withdrawal by the patient (*n *= 2) or during patient repositioning (*n *= 2), incorrect placement (*n *= 1), increased intracranial pressure (*n *= 1) and tube obstruction (*n *= 1). A total of 28 tubes (82%) were placed correctly in the jejunum. A gastric loop was detected by X-ray in 10 cases without adversely affecting enteral nutrition.

The procedure had to be aborted because of 1) increased intracranial pressure in a patient with head trauma during prolonged manipulation, 2) high residual gastric volume interfering with the pylorus visualization and 3) bleeding from gastric ulcers. In another three patients, X-ray showed incorrect prepyloric placement of the tube.

Three cases of bleeding occurred during the study and were examined by diagnostic endoscopy. An oesophageal mucosal defect was detected in one patient that required no further treatment. Ulceral bleeding was found in another two patients after the tube was indwelling for 3 and 15 days, respectively.

## Discussion

Our study examined the use of a new endoscope enabling the attending ICU physician to place jejunal feeding tubes transnasally independent of a special endoscopy team.

Transnasal endoscopy for the placement of postpyloric feeding tubes has already been described. It was either performed using a guidewire placed through the working channel of the endoscope [12-17] or by collecting the so far blindly inserted tube in the stomach with a forceps and subsequent advancement into the jejunum [18]. The success rate of the studies cited above ranged from 74.4% to 100% with the majority well above 90% and the procedure duration from 7.9 ± 3.8 minutes to 45 minutes. The procedures were carried out by endoscopists when reported.

In contrast to all previous studies we were able to advance the feeding tube directly through the working channel of the endoscope. In most of our patients the tube was positioned at first attempt.

Compared with other studies [16] the procedure time in our study is rather long. In our opinion this is compensated for by immediate availability of our procedure since it can be performed by the ICU physician. A learning effect can be expected with more experience.

There also exist approaches which attempt to place the feeding tubes without endoscopic guidance. These procedures require a certain degree of gastric emptying. Blind advancement of lubricated postpyloric feeding tubes with clockwise rotation was reported to achieve a 93% success rate when performed in the right lateral position after erythromycin use [19]. A success rate of 89% was achieved in another study when the tube placement was facilitated by external magnetic guidance [20]. A similar success rate of 88% was found when tubes with weighted ends and ECG guidance were used [21]. All these studies reported a mean procedure time of about 15 minutes. A shorter time interval of 7.8 minutes and a success rate of 80% were found in a study using the electromyography signal to identify the tube passage from the stomach to the duodenum [22]. Another study reported a success rate of 78% with spiral nasojejunal tubes compared with a rate of 14% with straight tubes, however, with a very low rate of correct positions [23].

Self-advancing tubes are an interesting alternative to all previous placement techniques. However, a low rate of successful tube placements was reported in patients with a high Simplified Acute Physiology Score (SAPS 2 [24]) [25]. Since the advancement of self-propelled tubes relies on gastric emptying and peristalsis, patients with high illness severity and pronounced gastrointestinal dysfunction may not benefit from the use of these tubes. Another drawback is the time delay of 2 to 68 hours until the correct position is reached [11]. This counterweighs the easiness of use as it impedes the early onset of enteral nutrition. An increased risk of mucosal damage was also reported [26].

Regarding the three cases of bleeding that occurred in our study, two of them were caused by ulcers. Whether the mucosal defect resulted from our procedure remains uncertain.

In summary, we believe that the placement of postpyloric tubes using endoscopy remains the most reliable option as impaired gastric emptying is the most frequent indication for jejunal feeding. All unguided procedures need adequate gastric emptying and self-advancing tubes do not guarantee the placement within 24 hours.

## Conclusions

The method described in this paper allows transnasal endoscopy and feeding tube placement at the bedside, which can be performed by an ICU physician. The procedure is safe and reliable, the success rate is good and complications are rare. As no endoscopist is needed, the implementation of this method facilitates early enteral nutrition. Rapid tube reinsertion after inadvertent displacement is also feasible.

## Key messages

• A new method for the placement of intestinal tubes for early enteral feeding is described.

• The method is easy to learn by intensivists.

• The method enables an early start of enteral nutrition.

## Abbreviations

ICU: Intensive care unit; SAPS: Simplified Acute Physiology Score.

## Competing interests

Gunnar Elke received lecture fees from Fresenius Kabi. All other authors declare that they have no competing interests.

## Authors' contributions

GZ participated in the design of the study, carried out the study and drafted the manuscript. AF, MA and BS carried out the study and participated in the analysis of data. GE, DS, IF, MS and NW participated in the analysis and interpretation of data. IF and GE revised the manuscript. NW conceived the study and participated in the design of the study, analysis and interpretation of data, and revision of the manuscript. All authors read and approved the final manuscript.
